# Identification and Functional Analyses of Host Proteins Interacting with the p17 Protein of Avian Reovirus

**DOI:** 10.3390/v14050892

**Published:** 2022-04-25

**Authors:** Chengcheng Zhang, Xinyi Liu, Fuxi Zhao, Qingqing Zhang, Wei Zuo, Mengjiao Guo, Xiaorong Zhang, Yantao Wu

**Affiliations:** 1 College of Veterinary Medicine, Yangzhou University, Yangzhou 225009, China; zcc@yzu.edu.cn (C.Z.); qtzdlxy99215@163.com (X.L.); ZFX951023@163.com (F.Z.); zqq1600676595@163.com (Q.Z.); zw15103447259@163.com (W.Z.); guomj@yzu.edu.cn (M.G.); zxr@yzu.edu.cn (X.Z.); 2 Jiangsu Co-Innovation Center for the Prevention and Control of Important Animal Infectious Disease and Zoonoses, Yangzhou 225009, China; 3 Comparative Medicine Research Institute, Yangzhou University, Yangzhou 225009, China

**Keywords:** avian reovirus, p17 protein, yeast two hybrid, protein interaction

## Abstract

Avian reovirus (ARV) causes viral arthritis, chronic respiratory diseases, retarded growth and malabsorption syndrome. However, the precise molecular mechanism remains unclear. Here, we report the host cellular proteins that interact with ARV p17 by yeast two-hybrid screening. In this study, the p17 gene was cloned into pGBKT7 to obtain the bait plasmid pGBKT7-p17. After several rounds of screening of a chicken cDNA library, 43 positive clones were identified as possible host factors that interacted with p17. A BLAST search of the sequences was performed on the NCBI website, which ultimately revealed 19 interacting proteins. Gene ontology enrichment and Kyoto Encyclopedia of Genes and Genome analyses indicated that the acquired proteins were involved in multicellular organismal processes, metabolic processes, and biological regulation. When the subcellular localization of the host protein and ARV p17 protein was investigated, we observed colocalization of p17-GFP with IGF2BP1-RED and PQBP1-RED in the transfected cells but not with FGF1-RED. The direct interaction of ARV p17 protein with IGF2BP1 and PQBP1 was confirmed by coimmunoprecipitation and GST pulldown assays. We used RT-qPCR to assess the expression variation during ARV infection. The results showed that IGF2BP1, PAPSS2, RPL5, NEDD4L, PRPS2 and IFI16 were significantly upregulated, whereas the expression of FGF1, CDH2 and PQBP1 was markedly decreased in DF-1 cells infected with ARV. Finally, we demonstrated that IGF2BP1 had a positive effect on ARV replication, while PQBP1 had the opposite effect. Our findings provide valuable information for better insights into ARV's pathogenesis and the role of the p17 protein in this process.

## 1. Introduction

Avian reovirus (ARV) belongs to the Orthoreovirus genus of Reoviridae, which are important pathogens that cause diseases including arthritis, malabsorption syndrome, immunosuppression and other chronic respiratory diseases in chickens [1,2,3]. ARVs cause considerable economic losses in the poultry industry [4].

As an icosahedral nonenveloped virus, the ARV genome consists of 10 double-stranded RNA segments that express 10 structural proteins and 4 nonstructural proteins [5], but the functions of many of these proteins are largely unknown. The S1 genome segment of avian reovirus contains three open reading frames and has been related to immunosuppression through interactions with host proteins that interfere with the innate immune response [6], one of which is the nonstructural protein p17 [7].

The nonstructural p17 protein of ARVs contains 146 amino acids (aa) and has been reported to play a critical role in virus–host interactions [8,9]. The p17 protein, which is known as a nucleocytoplasmic shuttling protein with a unique sequence [10,11] that specifically shuttles between the nucleus and the cytoplasm to regulate signaling pathways, including autophagy, gene transcription, and DNA binding, promotes viral replication and the cell cycle and interacts with several cellular proteins [11,12,13,14]. Several studies have reported that ARV p17 can inhibit cell growth and cause cell cycle retardation by initiating the p53 pathway in different cancer cell lines and interacting with cyclin-dependent kinases and different cyclins [11,15]. In contrast, the direct interaction of p17 with CDK1 leads to its inactivation and to suppression of the serine/threonine-protein kinase Plk1, which is important for a G2/M transition with oncogenic potential [16,17]. Moreover, the ARV p17 protein acts as a positive regulator to stabilize PTEN and enhance the Rak-PTEN interaction to prevent degradation of PTEN [18]. Recently, ARV p17 was reported to possess antiangiogenic activity by increasing the transcription and release of dipeptidyl peptidase 4, which is well known as a tumor suppressor molecule [19].

In this study, we identified the host proteins that interact with p17 from a cDNA library prepared from a chicken liver infected by the ARV GX/2010/1, and all the interactions between 19 host factors and p17 were reconfirmed by Y2H assays. We further examined the subcellular localization of some selected factors and assessed the expression of those proteins after ARV infection. Our study provides valuable information for better insights into ARV's pathogenesis and role of the p17 protein in this process.

## 2. Materials and Methods

### 2.1. Cells and Virus

DF-1 (an immortalized chicken embryo fibroblast cell line) and Vero (an African green monkey kidney cell line) cells were obtained from ATCC (USA) and cultured in Dulbecco’s modified Eagle’s medium (DMEM) (Life Technologies Corp., Grand Island, NY, USA) supplemented with 10% (vol/vol) fetal bovine serum (FBS), penicillin and streptomycin (50 IU/mL and 50 μg/mL, respectively, Sigma-Aldrich, Burlington, MA, USA) in a humidified atmosphere of 5% CO_2_. All cells were cultured until they reached 70–85% confluence before use. ARV strain GX/2010/1 (accession numbers KJ476699-KJ476708) isolated by our lab and propagated in Vero or DF-1 cells was used in the current research. After three freeze–thaw cycles, the supernatant was collected and stored at −70 °C. The virus titer of ARV was examined by plaque assay. Cells were infected with ARV at a multiplicity of infection (MOI) of 2.

### 2.2. Reagents

All restriction enzymes were purchased from NEB (USA). pEGFP-C1 and pDsRed-N1 vectors were obtained from Clontech (USA). TurboFect was purchased from Thermo Scientific. 40,6-Diamino-2-phenylindole (DAPI) was purchased from Beytime Company (Nanjing, China).

### 2.3. Cell Culture and Transfection

Vero cells were seeded in 6-well plates. Twenty-four hours later, when the cells had reached 70% confluence, the cells were transfected with the plasmids using the transfection reagent TurboFect according to the manufacturer's protocol.

### 2.4. Yeast Two-Hybrid Screen

The yeast two-hybrid screen was performed according to the manufacturer’s protocol (Clontech, Cat. No. 630489). The pGBKT7-p17 plasmid expressing the bait protein ARV p17 was transfected into competent Saccharomyces cerevisiae AH109 cells. A chicken cDNA library prepared from AVR-infected liver tissues using the pGADT7 plasmid for the fusion of proteins to GAL4-AD was introduced by transformation into competent Saccharomyces cerevisiae Y187 cells. Screening the interacting prey proteins by yeast mating was performed, as previously described [20]. Yeast cells transfected with pGADT7-T and pGBKT7-p53 or pGBKT7-Lam were used as positive and negative controls, respectively. Positive clones were transferred to quadruple dropout medium lacking tryptophan, leucine, histidine and adenine (SD/-Ade/-His/-Leu/-Trp, Clontech, CA, USA) and assessed by a PCR assay. The primers used are shown in Table 1. The results were sequenced and then subjected to a BLAST search against the NCBI database.

### 2.5. Functional Classification and Pathway Analysis

Functional classification analysis was performed using gene ontology and the UniProt database. Pathway analysis was performed mainly using the Kyoto Encyclopedia of Genes and Genomes (KEGG) database [21]. A protein interaction network was drawn based on the knowledge of the screened proteins. Based on the correlation between the proteins in the STRING 9.0 database [22], the host protein interaction network was constructed using Cytoscape v3.9.0 software.

### 2.6. Confocal Laser Scanning Microscopy Assay

For the subcellular localization assays, Vero cells were seeded on glass coverslips in 24-well plates and cultured overnight before transfection with pEGFP-p17 and pDsRed-PQBP1/IGF2BP1/FGF1. Thirty-six hours after transfection, the cells were fixed with 4% paraformaldehyde, and the nuclei were stained with Hoechst 33342 (10 ng/mL; Beyotime, Beijing, China). The images were observed using a laser confocal scanning microscope (LSM510 META; Zeiss, Germany).

### 2.7. Reciprocal Coimmunoprecipitation (co-IP) Assay

To further confirm whether the ARV p17 protein interacts with PQBP1, IGF2BP1 or FGF1, co-*IP* assays were carried out. After cotransfection of p17-Flag with recombinant vectors expressing PQBP1-Myc, IGF2BP1-Myc or FGF1-Myc for 48 h in DF-1 cells, immunoprecipitation was performed using an anti-c-Myc or anti-Flag agarose affinity gel (Thermo Fisher Scientific, 23620, Waltham, MA, USA) according to the manufacturer’s protocol. Briefly, cells were scraped from the culture plate and lysed with a mixture of RIPA buffer (Beyotime, Beijing, China) containing PMSF. After centrifugation for 20 min at 4 °C, the supernatant was collected, mixed with 10 μL of agarose slurry and incubated overnight at 4 °C. The protein agarose mixture was washed 3 times with TBST and resuspended in 2× nonreducing sample buffer. Finally, the liquid was heated for 5 min, with 2 μL of 2-ME added to the samples during denaturation, followed by analysis by Western blotting with specific antibodies.

### 2.8. GST Pulldown Assays

The procedure for GST pulldown was described in a previous study [15]. Briefly, GST or GST-p17 protein was expressed by induction in Escherichia coli BL21 (DE3) (Invitrogen, Carlsbad, CA, USA). Purified GST or GST-p17 protein (0.8 μg) was coupled to glutathione agarose (Thermo Scientific, 21516, Waltham, MA, USA), followed by washing with 1:1 wash solution (TBS (25 mM Tris-HCl, 0.15 M NaCl, pH 7.2), pull-down lysis buffer (with 10 μg/ml protease inhibitor mixture)) and incubated for 4 h at 4 °C with recombinant Myc-tagged host protein harvested from transfected Vero cells. The eluted proteins were denatured and examined by Western blot analysis with the corresponding antibodies.

### 2.9. Transfection with siRNA

PQBP1-, IGF2BP1- and FGF1-specific siRNA oligonucleotides and scrambled siRNA (negative control) were synthesized by GenePharma (Shanghai, China). The sequences used are shown in Table 1. DF-1 cells grown to 70% confluence were transfected with siRNA using TransIntro EL Transfection Reagent (TransGen Biotech, Beijing, China) in a 6-well plate. At 24 h post-transfection, one group of cells was collected, and the mRNA levels of specific proteins were assessed by qRT-PCR, while the remaining cells were infected with ARV at an MOI of 2; the protein levels of ARV p17 were analyzed by Western blotting after another 24 h.

### 2.10. Western Blot Assays

Vero or DF-1 cells were washed with PBS, collected, and lysed with radioimmunoprecipitation buffer containing the protease inhibitor PMSF (Santa Cruz, CA, USA). The lysates were centrifuged at 12,000 × g for 15 min at 4 °C. The concentration of solubilized protein in the supernatant was evaluated with the Bio-Rad Protein Assay (Bio-Rad Laboratories, Hercules, CA USA). The protein samples were mixed with 5× SDS–PAGE loading buffer and then boiled for 10 min at 100 °C. After electrophoresis at 100 V, proteins were transferred to a PVDF membrane by a semidry transfer instrument. Expression of the individual proteins was determined using the corresponding specific primary antibody and visualized by HRP-labeled secondary antibodies (diluted at 1:5000). The results were detected using a luminescent imager (Tanon 6600, Shanghai, China) after incubation of the membrane with enhanced chemiluminescence reagent (ECL plus) (Beyotime, P0018, Beijing, China). The intensity of the target protein was analyzed by ImageJ software.

### 2.11. Quantitative RT-PCR (qRT-PCR)

Total RNA was extracted from harvested DF-1 cells with or without ARV infection using an RNeasy Mini kit (Qiagen, Valencia, CA, USA). RNA quality was evaluated using RNA Nano Chips on an Agilent Bioanalyzer 2100 (Agilent Technologies, Santa Clara, CA, USA). Briefly, first strand cDNA was synthesized using 1 μg total RNA. Fivefold diluted cDNA products were used as templates for qRT-PCR by using a SYBR Green master mix (Takara, Dalian, China). The chicken β-actin gene was used as an internal reference to normalize the transcriptional value. All the primers used are listed in Table 1. The relative gene transcriptional levels were calculated using the 2^−^^ΔΔ^^CT^ method.

### 2.12. Overexpression of Target Protein Mediated by Recombinant Plasmids

The PQBP1-Myc, IGF2BP1-Myc and FGF1-Myc recombinant vectors encoding PQBP1, IGF2BP1 and FGF1, respectively, were used to study the effect of target protein overexpression on ARV replication. The recombinant plasmid was transfected into DF-1 cells using the TransIntro EL Transfection Reagent. After 24 h, the cells were infected with ARV. At 48 h post-infection, the viral genome copies were assessed by qRT-PCR.

### 2.13. MTT Assay

The cell viability after silencing PQBP1, IGF2BP1 and FGF1 was tested by an MTT method according to the manufacturer's instructions. Briefly, DF-1 cells were cultured in 96-well culture dishes. Different siRNAs were transfected when the cell confluence reached approximately 70%. After cultivation at 37 °C in an incubator with 5% CO_2_ for 24 h, the cell culture medium was removed, and 50 μL MTT (2 μg/mL) was added to each well. After incubation at 37 °C for 4 h, MTT was removed, and 200 μL DMSO was added. The plates were placed in an electronic oscillator for 10 min. Cell viability was quantified using a Multiskan FC Microplate Photometer (Thermo Scientific, Waltham, MA, USA).

### 2.14. Statistical Analysis

All experiments were conducted with at least three independent replicates, and all data analyses are expressed as the mean ± S.D. Statistical comparisons were made using the Student’s *t* test. Values of *p* < 0.05 were considered statistically significant.

## 3. Results

### 3.1. Identification of Cellular Proteins That Interact with the ARV p17 Protein through Screening of a Chicken cDNA Library

**Figure 1:**
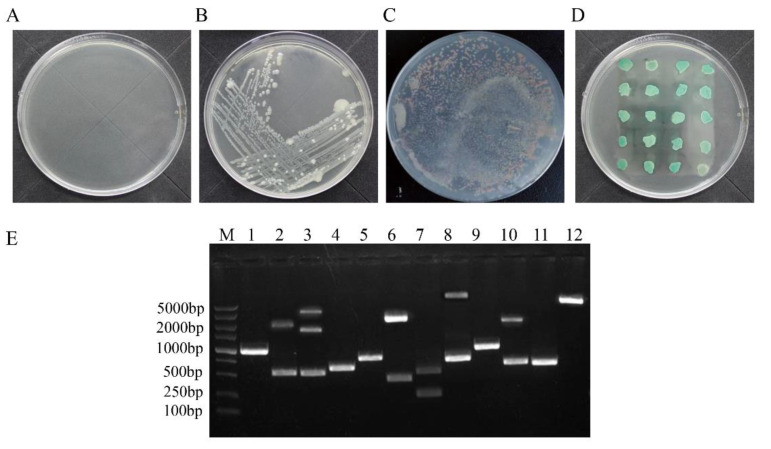
Screening for host proteins that interacted with ARV p17 in a chicken liver cDNA library by yeast two-hybrid assay. (**A**,**B**) show the negative and positive controls of the transformed competent cells grown on the SD/-His/-Leu/-Trp plate. (**C**) One of the transformed yeast strains cultured on SD/-His/-Leu/-Trp plates. (**D**) One of the SD/-His/-Leu/-Trp/-Ade/X-α-Gal plates on which the blue colonies indicate the positive proteins. (**E**) Identification of the positive clones. Plasmids were extracted from the blue colonies, which was used to amplify cDNA by PCR in the colony.

The cDNA library was constructed by using chicken liver cells infected with ARV GX2010/1 and then transformed into competent Y187 yeast cells. The pGBKT7-p17 plasmid was transformed into competent yeast Y2H Gold cells, which were then mixed with 1 ml of the Y187 cells that contained cDNA library and cultured at 30 °C and 50 rpm for 20–24 h. The AH109 cells containing the negative or positive control plasmid indicated that the cotransformation process was successful (Figure 1A,B). Then, the cells were spread onto quadruple dropout medium plates and cultivated at 30 °C until colonies appeared. The results are shown in Figure 1C. For the screened transformants, 65 clones grew on the quadruple dropout medium plates. After 3 repeated inoculations on the quadruple dropout medium plates, 43 blue colonies were considered positive candidate proteins (Figure 1D).

### 3.2. Positive Protein Confirmation and Sequence Analysis

As shown in Figure 1D, out of 43 isolated yeast colonies, 35 were reconfirmed as positive after a second cotransformation with bait plasmid into the Y2H competent cells. The positive samples were sequentially tested by PCR for the library plasmids, and the screened cDNAs from the library were primarily between 500 and 2000 bp in length (Figure 1E). The sequences were then aligned with the basic local alignment search tool (BLAST) on the NCBI website against chicken nonrefSeq databases (Table 2). The BLAST results indicated that there were 19 interacting proteins: ribose-phosphate pyrophosphokinase 2 (PRPS2), gamma-interferon-inducible protein 16 (IFI16), nucleolar GTP-binding protein 1 (GTPBP4), polyglutamine-binding protein 1 (PQBP1), insulin-like growth factor 2 mRNA-binding protein 1 (IGF2BP1), neutral cholesterol ester hydrolase 1 (NCEH1), fibroblast growth factor (FGF1), 3′-phosphoadenosine-5′-phosphosulfate synthase (PAPSS2), cadherin-2 (CDH2), Phasianus colchicus ribosomal protein L5 (RPL5), Coturnix japonica ribosomal protein L7 (RPL7), Gallus gallus discs large MAGUK scaffold protein 1 (DLG1), Gallus gallus dehydrogenase/reductase 3 (DHRS3), Phasianus colchicus NEDD4 like E3 ubiquitin protein ligase (NEDD4L), Gallus gallus FERM domain containing 8 (FRMD8), Gallus gallus zinc finger protein 598 (ZNF598), hypothetical protein and 2 predicted proteins.

### 3.3. Functional Classification and Pathway Analysis

**Figure 2:**
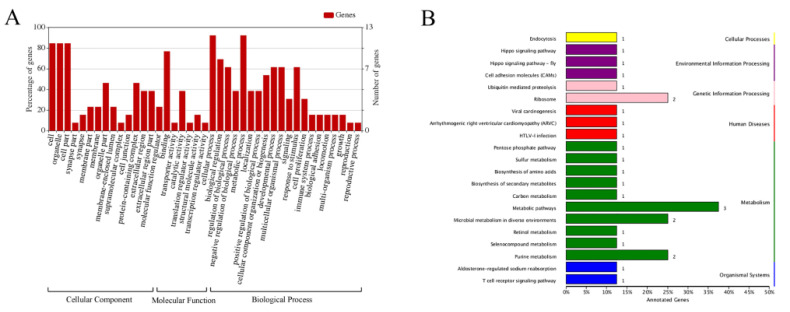
Functional classification and pathway analysis of the screened proteins. (**A**) The classification of the screened proteins was performed according to GO biological processes. All GO categories with a *p* value were chosen. The vertical axis is the percentage of genes, and the horizontal axis is the GO functional category. (**B**) Kyoto Encyclopedia of Genes and Genomes (KEGG) pathway enrichment analyses for the 19 host proteins interacting with ARV p17.

After identifying the 19 host proteins that interacted with the ARV p17 protein, GO and KEGG databases were used to identify the main functional groups of the acquired genes. The 19 identified proteins were divided into 41 functional groups, which belonged to biological processes (BP), cellular components (CC), and molecular functions (MF). Multicellular organismal processes, metabolic processes, and biological regulation were the majority of the BP terms. The main subcategories in the MF category were catalytic activity and translation regulators. The five main CC subcategories were cell, organelle, cell parts, protein-containing complex and bindings (Figure 2A). In addition, 26 KEGG pathways were obtained for the identified proteins. The pathways of metabolism, genetic information processing, environmental information processing and diseases were associated with the highest numbers of identified proteins (Figure 2B).

### 3.4. Construction of the ARV p17-Cellular Protein Interaction Network

**Figure 3:**
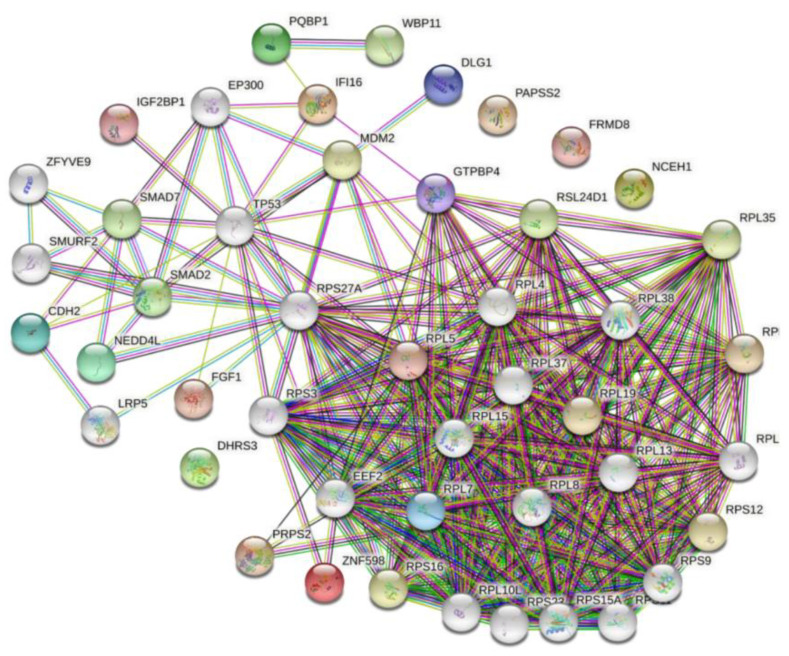
The network of cellular proteins. The network of host proteins interacting with ARV p17 was constructed based on the STRING 9.0 database. GTPBP4, RPL5, RPL7, IFI16, and PRPS2 were the most remarkable node proteins in the interaction network of the cellular factors.

In the p17-cellular protein interaction network (Figure 3), GTPBP4, IFI16, and PRPS2 were the most remarkable node proteins, whereas PQPB1, IGF2BP1, FGF1, CDH2, DLG1, NEDD4L and ZNF598 were less remarkable node proteins.

### 3.5. ARV p17 Protein Colocalizes with PQBP1/IGF2BP1 in Host Cells

**Figure 4:**
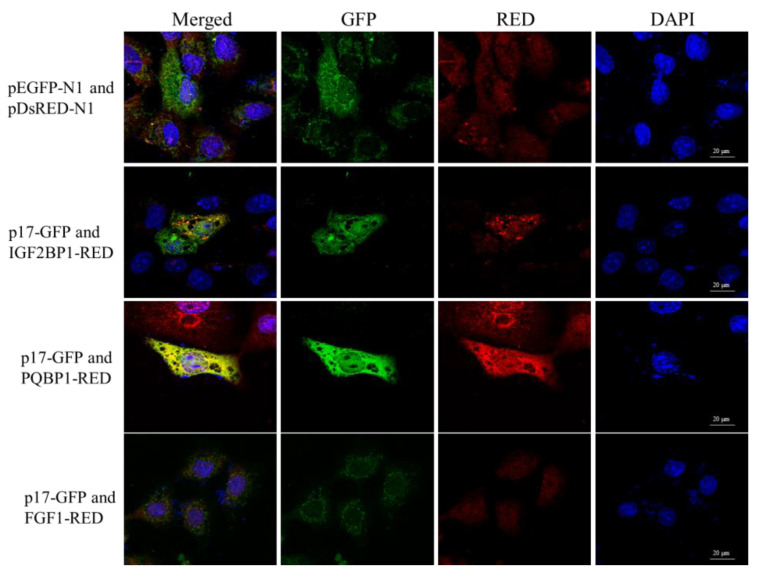
The colocalization of ARV p17 with the screened host proteins. Vero cells were cotransfected with p17-GFP and IGF2BP1, PQBP2 or FGF1-RED and then analyzed by laser confocal microscopy after 36 h. All cells were stained with Hoechst 33342.

To determine the subcellular colocalizations of the identified ARV p17-interacting host factors, we performed a confocal microscopy assay by cotransfecting three proteins as fusions to the N-terminus of RED (PQBP1-RED/IGF2BP1-RED/FGF1-RED) with p17-GFP in Vero cells (Figure 4). When cells were transfected with both plasmids, we observed the p17-GFP, IGF2BP1-RED, PQBP1-RED and FGF1 all location both in the cytoplasm and the nucleus in the transfected cells. However, the colocalization phenomenon exists between p17-GFP with IGF2BP1-RED and PQBP1, but not with FGF1-RED (Figure 4).

### 3.6. ARV p17 Protein Interacts with PQBP1/IGF2BP1 In Vivo and In Vitro

**Figure 5:**
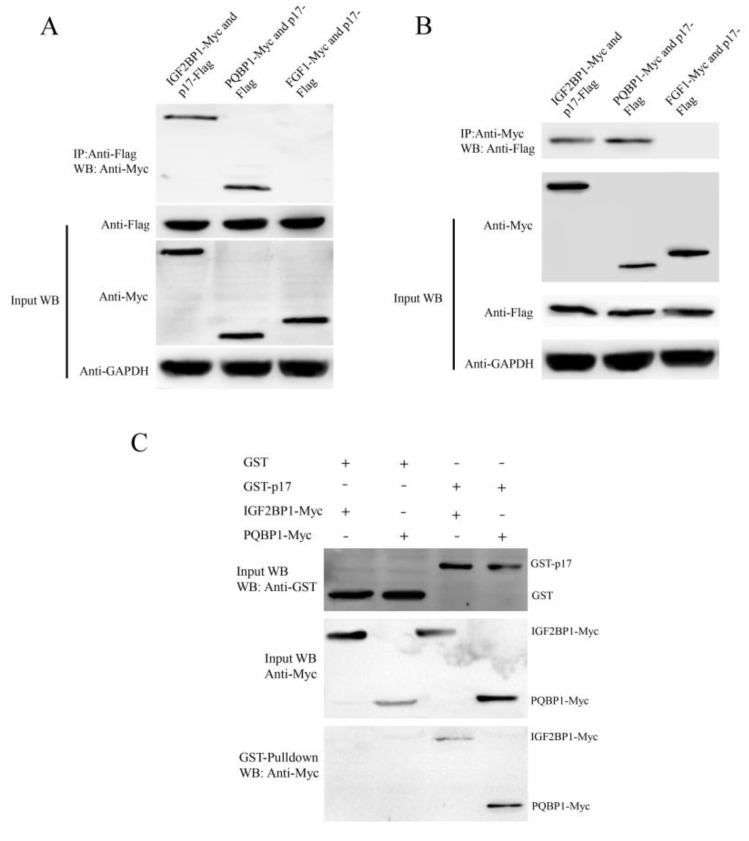
The ARV p17 protein interacts with PQBP1/IGF2BP1. (**A**) A coimmunoprecipitation assay demonstrated that p17-Flag bound to IGF2BP1-Myc and PQBP1-Myc but not to FGF1-Myc in cotransfected cells. Vero cells were transfected with p17-Flag and IGF2BP1-Myc or PQBP1-Myc plasmids for 36 h and then harvested. Cell lysates were immunoprecipitated with an antibody against Flag, followed by Western blotting analysis. (**B**) Reciprocal co-IP experiments showed that the anti-Myc antibody precipitated p17-Flag. (**C**) GST pulldown assay. Glutathione beads conjugated to GST or the GST-p17 fusion protein were incubated with recombinant IGF2BP1-Myc or PQBP1-Myc protein. After washing, proteins were eluted from the beads. The IGF2BP1-Myc and PQBP1-Myc proteins were assessed by immunoblotting with an anti-Myc mAb. GST and GST-LC3 protein expression was confirmed by immunoblotting with a rabbit anti-GST pAb.

Because the ARV p17 protein colocalized with PQBP1/IGF2BP1, coimmunoprecipitation and GST pulldown assays were performed to further verify the interaction between p17 and PQBP1/IGF2BP1. The p17-Flag plasmid was cotransfected with those expressing PQBP1-Myc, IGF2BP1-Myc and FGF1-Myc in Vero cells for 24 h, and the cell lysates were coimmunoprecipitated with anti-Flag or anti-Myc affinity gel. After the co-IP process, the proteins in the complexes were assessed by Western blotting. As shown in Figure 5, the ARV p17 protein could interact with IGF2BP1 and PQBP1 but not with FGF1.

The GST pull-down assay was performed to confirm the interaction between p17 and PQBP1/IGF2BP1 in vitro. Recombinant full-length p17-GST fusion protein and GST protein were mixed with the proteins PQBP1-Myc or IGF2BP1-Myc expressed in Vero cells and incubated at 4 °C overnight. As shown in Figure 5, PQBP1-Myc and IGF2BP1-Myc were detected in a complex with p17-GST. These results illustrate that p17 can interact with PQBP1/IGF2BP1 in vitro.

### 3.7. The mRNA Expression of Interacting Proteins after ARV Infection

**Figure 6:**
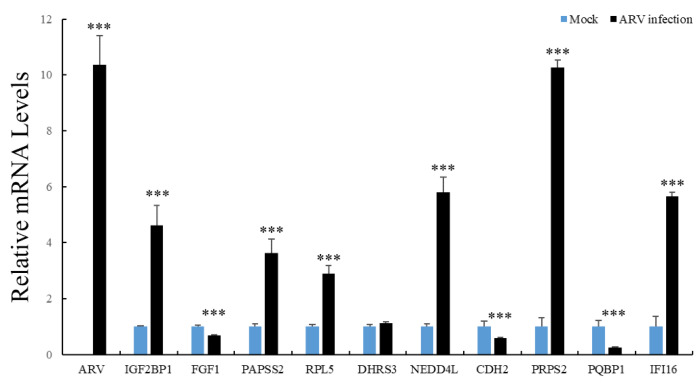
Detection of the mRNA expression of the identified protein interacting with ARV p17 after ARV infection for 12 h by qRT-PCR. Data were pooled across experiments and analyzed using *t* tests. Bars indicate the grand means ± standard deviation (SD). ***, *p* < 0.001.

To study whether the identified host factors play a role in viral replication, the mRNA expression levels of 10 interacting proteins were assessed by using qRT-PCR analysis in ARV-infected DF-1 cells. The results showed that the transcriptional level of IGF2BP1, PAPSS2, RPL5, NEDD4L, PRPS2 and IFI16 were significantly upregulated after ARV infection for 12 h, whereas FGF1, CDH2 and PQBP1 were significantly downregulated (Figure 6).

### 3.8. The Selected Proteins Have Different Effects on Viral Replication

**Figure 7:**
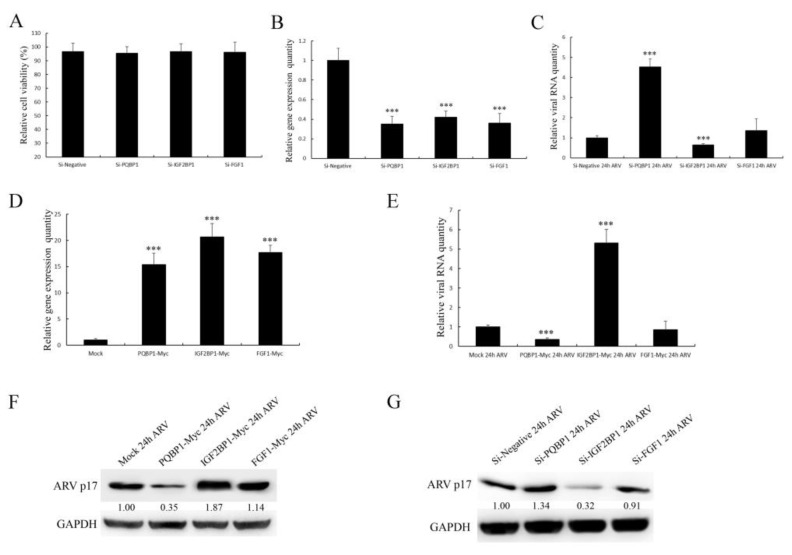
The effect of the selected proteins on ARV replication. (**A**) An MTT assay assessed cell viability. (**B**) Knockdown of IGF2BP1, PQBP1 and FGF1 mediated by siRNA. (**C**) The replication level of ARV assessed by qRT–PCR. (**D**) Overexpression of IGF2BP1, PQBP1 and FGF1 mediated by plasmids. (**E**) The replication level of ARV assessed by qRT–PCR. (**F**,**G**) The replication level of ARV assessed by Western blotting. Data in the graph represent the mean ± S.D. (error bars) calculated from three independent experiments. ***, *p* < 0.001. Signals for all blots were quantified using ImageJ software.

To further study the effect of selected proteins on ARV replication, IGF2BP1, PQBP1 and FGF1 were successfully overexpressed or knocked down in DF-1 cells, as shown in Figure 7B,D. Then, the transfected cells were infected with ARV for 36 h. The qRT-PCR and WB results showed that IGF2BP1 had a positive effect, while PQBP1 had an inhibitory effect on ARV proliferation (Figure 7C,E–G). Under the same conditions, FGF1 showed no significant effect on ARV replication.

## 4. Discussion

ARV causes viral arthritis, chronic respiratory diseases, retarded growth and malabsorption syndrome, leading to considerable losses to the poultry industry. To successfully infect cells, viruses always tend to manipulate or utilize many host factors through directly interactions. The ARV p17 protein is also a multifunctional protein that regulates signaling pathways, including autophagy, gene transcription, DNA binding and the cell cycle, and interacts with several cellular proteins [11,12], but its molecular mechanism is largely unknown. The identification of interacting host factors will provide insights into the p17 protein-mediated viral process. In the current study, we identified 19 host proteins that can interact with ARV p17. The interactions of p17 with IGF2BP1 and PQBP1 were verified by co-IP and GST pulldown assays (Figure 5). From our research, IGF2BP1 was shown to play a positive role in ARV replication, while PQBP1 was shown to play an opposite role (Figure 7). Through these host factors, we can further study the mechanism of the function of ARV p17 and the pathogenesis of ARV infection.

IFI16 belongs to the IFN-inducible PYHIN-200 gene family, which contains the two signature HIN domains that bind to dsDNA or ssDNA and a PYRIN domain that mediates interactions with proteins [23,24,25]. Ribose phosphate pyrophosphate kinase 2 (PRPS2) is a key enzyme in the synthesis of 5-phosphate ribose 1-pyrophosphate (PRPP), an important raw material for DNA synthesis, and plays a critical role in promoting cell apoptosis and inhibiting cell proliferation [26]. Gtp-binding protein 4 (GTPBP4), a member of the GTPBPS family, is a novel G protein located in the nucleolus that mainly participates in the synthesis and maturation of 60S subunits, which are closely related to cell proliferation and growth [27]. Polyglutamine binding protein 1 (PQBP1), a nuclear protein expressed mainly in lymphoid and myeloid cells, can bind to proteins containing poly Q regions and participate in the transcription and RNA modification process, which has been shown to lead to the production of type I interferon in retrovirus infection [28]. Insulin-like growth factor 2 mRNA binding protein 1 (IGF2BP1) is a component of messenger ribonucleoprotein particles, a conserved single-stranded RNA-binding protein family that mediates the transcription of β-actin mRNA and related proteins, regulates cell metabolism and promotes cell adhesion and survival [29]. Fibroblast growth factor 1 (FGF1), a member of the growth factor family, plays an important role in embryonic development, vascular growth, wound healing and other processes [30]. 3'-Adenosine-5'-phosphosulfate synthase 2 (PAPSS2) is an important enzyme gene that catalyzes the synthesis of active sulfate donors in vivo, and its activity is important for normal bone development [31]. Cadherin 2 (CDH2) is a class of calcium-dependent cell adhesion molecules that regulate cell–cell adhesion through homozygous binding in fixed epithelial tissues [32].

Studies that include screening host factors interacting with ARV proteins are relatively rare. In our study, we observed the colocalization of p17-GFP with IGF2BP1-RED and PQBP1-RED both in the cytoplasm and the nucleus in the transfected cells. However, FGF1-RED did not colocalize with ARV p17-GFP (Figure 5). The various distributions of the screened host proteins implied that the ARV p17 protein is indeed a multifunctional protein involved in distinct cellular pathways. As shown in the network of cellular proteins, GTPBP4, FGF1 and IFI16 interacted with ARV p17 but were also involved in interactions with other signaling pathway proteins (Figure 4). These notable node proteins tended to be more essential than the other less notable proteins in the regulation and influence of p17-mediated functions. As shown in Figure 5, the ARV p17 protein interacts with PQBP1/IGF2BP1 verified by co-IP and GST-pulldown assays. PQBP1 is essential to induce the nuclear translocation of nuclear factor κB (NFκB), NFκB-dependent transcription of inflammation genes, brain inflammation in vivo, and eventually mouse cognitive impairment. As shown in Figure 7, PQBP1 had an inhibitory effect on ARV proliferation; interestingly, ARV p17 can interact with PQBP1, and those interactions may play a key role in regulating PQBP1’s function.

Several host proteins have been shown to specifically interact with structural ARV proteins. In most of these cases, these interactions play a critical role in adjusting virus replication and, moreover, may be involved in host cell fate or viral virulence [33,34]. The identified host proteins that interact with ARV p17 increase our understanding of the molecular mechanism of ARV infection. We believe that further study of these host proteins and their relationship with p17 can provide valuable information for better insights into ARV's pathogenesis and the role of the p17 protein in this process.

## Figures and Tables

**Table 1:** Sequence of PCR primers.

Gene Name	Primer Sequence (5’-3’)	Note	Accession Number
AD-F AD-R	TAATACGACTCACTATAGGGCT GTGAACTTGCGGGGTTTTTCAGTATCTACGATT	Amplification of cDNA inserted in AD plasmid	
p17-GFP-F	GCGAATTCTATGCAATGGCTCCGCCATACG	Amplification of ARV p17 gene with GFP label	
p17-GFP-R	GCGGATCCCTCATGGATCGGCGTCAAATCG		
p17-Flag-F	ATTGGATCCTATGCAATGGCTCCGCCATACG	Amplification of ARV p17 gene with Flag tag Amplification of ARV p17 gene with GST label	
p17-Flag-R p17-GST-F p17-GST-R	ATTCTCGAGTCACTTATCGTCGTCATCCTTGTAATCCTCATGGATCGGCGTCAAATCG GCGAATTCTATGCAATGGCTCCGCCATACG GCCTCGAGCTCATGGATCGGCGTCAAATCG		
PQBP1-RED-F	CGGAATTCATATGCCGCTGCCCGTTG	Amplification of PQPB1 gene with RED label Amplification of PQBP1 gene with Myc tag	AJ973596.1
PQBP1-RED-R PQBP1-Myc-F PQBP1-Myc-R	CGGGATCCACCTGCTGCTTGGTT ATTGGATCCATGCCGCTGCCCGTTG ATTCTCGAGTCACAGATCCTCTTCAGAGATGAGTTTCTGCTCACCTGCTGCTTGGTT		
IGF2BP1-RED-F	CGGAATTCCCGTTGCTGTCGGG	Amplification of IGF2BP1 gene with RED label Amplification of IGF2BP1 gene with Myc tag Amplification of FGF1 gene with RED label Amplification of FGF1 gene with Myc tag	NM_205071.1
IGF2BP1-RED-R IGF2BP1-Myc-F IGF2BP1-Myc-R FGF1-RED-F FGF1-RED-F FGF1-Myc-F FGF1-Myc-R	CGGGATCCGTTCTTAGCCCCAT ATTGGATCCATGCCGTTGCTGTCGGG ATTCTCGAGTCACAGATCCTCTTCAGAGATGAGTTTCTGCTCGTTCTTAGCCCCAT CGGAATTCATGGCCGAGGGGG CGGGATCCGGCACGCTTGGATC ATTGGATCCATGGCCGAGGGGG ATTCTCGAGTCACAGATCCTCTTCAGAGATGAGTTTCTGCTCGGCACGCTTGGATC		KY747397
IGF2BP1-F	AAGGCACAAGGCAGGATT	Detect the expression of IGF2BP1 by RT qPCR	NM_205071.1
IGF2BP1-R	GCAGCTCATTGACGGTTTT		
FGF1-F	AAAAGCACGCAGACAAGAAC	Detect the expression of FGF1 by RT qPCR	KY747397
FGF1-R	CATTGGAACACCAGGAAGG		
PAPSS2-F	TTGATGCAGGACACTCGC	Detect the expression of PAPSS2 by RT qPCR	XM_040674794
PAPSS2-R	CAATGGTTGACTTGGGAT		
RPL5-F RPL5-R	TTTCCCTGGTTATGACTC AGCATCTTCATCCTCCTC	Detect the expression of RPL5 by RT qPCR	XM_031614822.1
DHRS3-F DHRS3-R	ATGCCTGTTGAGGTCTGC AAAGTTTGGTGGAGTGGA	Detect the expression of DHRS3 by RT qPCR	NM_001277910.3
NEDD4L-F NEDD4L-R	TGCGGATAGCACCCAATG TCCTTTCCTCCCAACCAG	Detect the expression of NEDD4L by RT qPCR	XM_031609223.1
CDH2-F CDH2-R	ATCCTACTGGACGGTTCG TTGGCTAATGGCACTTGA	Detect the expression of CDH2 by RT qPCR	NM_001001615
PRPS2-F PRPS2-R	CGTGGAGCGTTGGAGTCGT GCGGGTTCTGCATACAGGTTAT	Detect the expression of PRPS2 by RT qPCR	NM_001006264.1
PQBP1-F PQBP1-R IFI16-F IFI16-R	TGCCGAGGACTATGACGA AGCTTCTTGGCCGATTTG CTGGAACGAAAGGGAG TTGGGTGGAGCTGAT	Detect the expression of PQBP1 by RT qPCR Detect the expression of IFI16 by RT qPCR	AJ973596.1 NM_001131692.1
ARV-F ARV-R	CGTATCATTCACCCGCGATT TGTTCGCTGTACCATCACCT	Detect the replicaiton of ARV by RT qPCR	
GAPDH-F GAPDH-R Si-PQBP1 Si-IGF2BP1 Si-FGF1 Si-Negative	GGTGGTGCTAAGCGTGTTA CCCTCCACAATGCCAA UGGCCAAGAGAGGCAUCCUCAAACA CAGUGGGCCAUGAAAGCCAUCGAAA CGGAAGAUGUGGGCGAGGUCUAUAU UUCUCCGAACGUGUCACGUUU	Detect the expression of GAPDH by RT qPCR SiRNA target of PQBP1 SiRNA target of IGF2BP1 SiRNA target of FGF1 Negative control	AJ973596.1 NM_205071.1 KY747397

The underline sequences represent the restriction enzyme cutting site.

**Table 2:** The positive proteins used for yeast two-hybrid analysis.

No.	GenBank	Protein Name	Description	ORF (bp)
1	NM_001006264.1	Ribose-phosphate pyrophosphokinase 2 (PRPS2)	Activated by magnesium and inorganic phosphate. Competitively or non-competitively inhibited by ADP, or GDP.	1231
2	NM_001131692.1	Gamma-interferon-inducible protein 16 (IFI16)	A predominantly nuclear protein involved in transcriptional regulation, also functions as an innate immune response DNA sensor and induces the IL-1β and antiviral type-1 interferon-β (IFN-β) cytokines.	3012
3	NM_001006354.1	Nucleolar GTP-binding protein 1 (GTPBP4)	Nucleolar GTP-binding protein 1 is a protein that in humans is encoded by the GTPBP4 gene.	1983
4	AJ973596.1	Polyglutamine-binding protein 1 (PQBP1)	A protein predominantly expressed in lymphoid and myeloid cells was initially identified in the pre-mRNA splicing by interacting with splicing-related factors.	1133
5	NM_205071.1	Insulin-like growth factor 2 mRNA-binding protein 1 (IGF2BP1)	It serves as a post-transcriptional fine-tuner regulating the expression of some essential mRNA targets required for the control of tumor cell proliferation and growth, invasion, and chemo-resistance, and metastasis in various types of human cancers.	2021
6	XM_040679547	Neutral cholesterol ester hydrolase 1 (NCEH1)	Hydrolysis of intracellular CE is the rate-limiting step in the cholesterol efflux from macrophage foam cells. As the hydrolysis of CE takes place at neutral pH, the enzymes catalyzing it have been collectively called neutral CE hydrolases (NCEHs).	5520
7	KY747397	Fibroblast growth factor (FGF1)	Fibroblast growth factors (FGFs) that signal through FGF receptors (FGFRs) regulate a broad spectrum of biological functions, including cellular proliferation, survival, migration, and differentiation.	556
8	XM_040674794	3'-phosphoadenosine-5'-phosphosulfate synthase (PAPSS2)	Blood clotting, bone development 3'-Adenosine phosphate-5'-Sulphate biosynthesis	6730
9	NM_001001615	Cadherin-2 (CDH2)	This protein plays a role in the establishment of left-right asymmetry, development of the nervous system and the formation of cartilage and bone.	3203
10	XM_031614822.1	Phasianus colchicus ribosomal protein L5 (RPL5)	The encoded protein binds 5S rRNA to form a stable complex called the 5S ribonucleoprotein particle (RNP), which is necessary for the transport of non-ribosome-associated cytoplasmic 5S rRNA to the nucleolus for assembly into ribosomes.	1034
11	XM_015855784.2	Coturnix japonica ribosomal protein L7 (RPL7)	The protein has been shown to be an autoantigen in patients with systemic autoimmune diseases. As is typical for genes encoding ribosomal proteins, there are multiple processed pseudogenes of this gene dispersed through the genome.	921
12	XM_025153616.1	Gallus gallus discs large MAGUK scaffold protein 1 (DLG1)	This gene encodes a multi-domain scaffolding protein that is required for normal development. This protein may have a role in septate junction formation, signal transduction, cell proliferation, synaptogenesis and lymphocyte activation.	4586
13	NM_001277910.3	Gallus gallus dehydrogenase/reductase 3 (DHRS3)	DHRS3 (dehydrogenase/reductase 3) is a protein coding gene. Diseases associated with DHRS3 include amphetamine abuse and neuroblastoma. Among its related pathways are vitamin A and carotenoid metabolism and metabolism of fat-soluble vitamins.	4798
14	XM_031609223.1	Phasianus colchicus NEDD4 like E3 ubiquitin protein ligase (NEDD4L)	NEDD4Lis a protein coding gene. Diseases associated with NEDD4L include periventricular nodular heterotopia 7 and periventricular nodular heterotopia.	7445
15	XM_040694238.1	Gallus gallus FERM domain containing 8 (FRMD8)	It has confirmed the pathophysiological significance of FRMD8 in iPSC-derived human macrophages and mouse tissues, thus demonstrating its role in the regulated release of multiple cytokine and growth factor signals.	9815
16	XM_015294442	Gallus gallus zinc finger protein 598 (ZNF598)	It plays an important role in regulating gene expression, cell differentiation, embryo development and other life processes.	2797
17	AJ720578.1	Gallus gallus mRNA for hypothetical protein	Hypothetical protein	838
18	XR_004306526.1	Coturnix japonica uncharacterized LOC116653138	Predicted protein	2760
19	XM_025145628.1	Gallus gallus NADH-ubiquinone oxidoreductase chain 1-like LOC112530942	Predicted protein	1821

## Data Availability

The data presented in this study are available in the article.
